# Worm-Based Alternate Assessment of Probiotic Intervention against Gut Barrier Infection

**DOI:** 10.3390/nu11092146

**Published:** 2019-09-08

**Authors:** Juil Kim, Yuseok Moon

**Affiliations:** 1 Laboratory of Mucosal Exposome and Biomodulation, Department of Biomedical Sciences and Biomedical Research Institute, Pusan National University, Yangsan 50612, Korea; 2 College of Information and Biomedical Engineering, Pusan National University, Yangsan 50612, Korea

**Keywords:** gut health, probiotics, *Caenorhabditis elegans*, epithelial barrier infection

## Abstract

The epithelial barrier is the frontline defense against enteropathogenic bacteria and nutrition-linked xenobiotic stressors in the alimentary tract. In particular, enteropathogenic *Escherichia coli* (EPEC) insults the gut barrier and is increasingly implicated in chronic intestinal diseases such as inflammatory bowel disease. For the efficient development of intervention against barrier-linked distress, the present study provided a *Caenorhabditis elegans*-based assessment instead of extensive preclinical evaluations using mammalian models. In particular, EPEC infected the gut and shortened the lifespan of *C. elegans*, which was counteracted by colonization of *E. coli* strain Nissle 1917 (EcN). In addition to the competitive actions of EcN against EPEC, EcN improved the gut barrier integrity of worms via the Zonula occludens ortholog (Zoo-1) induction, which was verified in the murine infection and colitis model. The worm-based assessment provided a crucial methodology and important insights into the potent chronic events in the human gut barrier after the ingestion of probiotic candidates as a mucoactive dietary or therapeutic agent.

## 1. Introduction

*Escherichia coli* strain Nissle 1917 (O6:K5:H1, EcN) is a nonpathogenic fecal bacterium with probiotic activities in humans and animals [1,2,3]. This strain was originally isolated from the gut of a German soldier who displayed resistance to endemic shigellosis during World War I, unlike his comrades [4]. Moreover, limited clinical investigations using *E. coli* Nissle 1917 have demonstrated that probiotic-based therapeutic applications can be efficacious in patients with chronic ulcerative colitis [5,6,7] and irritable bowel syndrome [8]. *E. coli* Nissle 1917 is relatively safe for therapeutic applications since it does not cause colitis, even in gnotobiotic animals that are mono-inoculated with the strain [9]. In terms of molecular genetics, *E. coli* Nissle 1917 does not produce any virulence factors or carry any genes for pathogenicity traits and does not form enterotoxins, cytotoxins, or hemolysins [10,11]. Thus, this supports the general recognition of *E. coli* Nissle 1917 as a safe organism for human use. In terms of infectious diseases, EcN treatment can attenuate cell death of *Salmonella*-infected epithelial cells. Moreover, it can activate the synthesis of defensins and zonula occludens protein-1 (ZO-1), which is a tight junction (TJ) protein [12].

The gut epithelial barrier is the front layer that is first encountered by dietary components and is an essential line of defense against the detrimental luminal environment, including pathogens and xenobiotics. Thus, this is important for maintaining the host integrity. The disruption of the intestinal barrier is closely associated with microbial invasion and subsequent systemic spreading of pathogens, which results in widespread toxicity [13]. The intestinal epithelial barrier is composed of epithelial cells and junctional complexes, such as adherence junctions, gap junctions, desmosomes, and TJs. The disruption of these complexes allowed invasion by gut luminal microbes and subsequent stimulation of submucosal immune responses. Barrier disruption-associated bacterial invasion is the main cause of the pro-inflammatory insults in the chronic ulcerative diseases, including Crohn’s disease (CD) and ulcerative colitis (UC). Moreover, mucosa-associated *E. coli* strains, including enteropathogenic *E. coli* (EPEC), are frequently observed on the intestinal surface of patients with chronic diseases, such as inflammatory bowel disease (IBD) and colorectal cancer [14,15,16,17]. Although the pathophysiology of enteropathogenic EPEC-induced diarrhea remains unclear, numerous studies have addressed the pathogen-specific effects on host epithelial cells [18,19,20]. Therefore, efficient epithelial barrier-protective interventions need to be developed using the competitive probiotic bacteria-based food materials against the gastrointestinal distress and other involved factors, such as mucosa-associated *E. coli*.

As the simplest model of a mammalian intestine, the gut of *Caenorhabditis elegans* can simulate the epithelial response to the luminal factors. Since *C. elegans* lacks any identified ‘professional’ leukocytes, such as macrophages and lymphocytes to defeat pathogens, it depends on the gut epithelial barrier for immunity [21,22]. Moreover, the epithelium-based defense in invertebrates, such as *C. elegans,* is crucial in the maintenance of their biological integrity during their lifespan [21,23,24,25]. With many practical advantages, experiments with *C. elegans* do not raise any of the ethical concerns associated with the use of mammals. Furthermore, *C. elegans* represents a multicellular organism that is a self-fertilizing hermaphrodite. It has a high progeny rate, a short life cycle, and can be easily maintained in the laboratory [26,27].

For the efficient development of mucoactive probiotic bacteria, an extensive preclinical analysis of the candidate bacteria is needed using the animal gut exposure models. However, in terms of the regulation in animal welfare and ethics, a *C. elegans*-based gut could be used as an alternative model for the assessment of the gut barrier exposed to the probiotic candidates. Moreover, due to the diversity and wealth of its genomic information, it is convenient to use *C. elegans* to understand mechanisms of mammalian epithelial barrier-associated immunity. In the present study, we evaluated the *C. elegans*-based gut exposure model to assess the barrier action of probiotics, such as EcN. Since the gut epithelium is the important frontline for the recognition of new food factors, the simplest epithelium of *C. elegans* would provide a valuable platform for good extrapolations to the probiotic actions of valuable dietary components in the human gut.

## 2. Materials and Methods

### 2.1. C. elegans Strains and Culture Conditions

*C. elegans* Bristol N2 (Brenner 1974) (*Caenorhabditis* Genetics Center, University of Minnesota, Minneapolis, MN, USA) was maintained at 20–25 °C on nematode growth medium (NGM) agar (50 mM NaCl, 1.7% agar, 0.25% peptone, 1 mM CaCl_2_, 5 μg/mL of cholesterol, 1 mM MgSO_4_ and 25 mM KPO_4_ in dH_2_O) plates spread with *E. coli* OP50 (Pohang, South Korea) or EcN as a food source. *C. elegans* was synchronized with a mixture of 500 μL of 5 M NaOH, 1 mL of 5% solution of sodium hypochlorite (Yuhan-Clorox, Seoul, South Korea), and 3.5 mL of autoclaved dH_2_O. Synchronized eggs were seeded on the NGM plate for growth while the worms at L4 stage were seeded on a new NGM plate with or without 50 µM 5-fluoro-2′-deoxyuridine (FUdR, Tokyo Chemical Industry, Portland, OR, USA). *E. coli* OP50 and EcN (OD_600_ = approximately 0.6–0.8) were spread on this dish. After this, worms were exposed to EPEC (OD_600_ = approximately 0.6–0.8) for the time indicated. For the lifespan assays in the presence of each bacterium, presynchronized L4 worms were grown on the *E. coli* OP50, EcN, or EPEC lawn (without tryptophan) for 48 h. For lifespan assays to measure the impact of EcN pretreatment, presynchronized L4 worms were grown on the *E. coli* OP50 and EcN lawns for 48 h before being grown with EPEC on NGM plates containing tryptophan (2 mg/mL) for 48 h. To prevent the produced offspring from producing progeny, worms were transferred to a new NGM plate every day. The survival of worms was scored every day, which involved determining their ability to move in response to touch with a platinum wire.

### 2.2. RNA Preparation, Reverse Transcription, and Real-Time Quantitative PCR (qPCR)

About 1000 *C. elegans* were collected using M9 buffer (42 mM Na_2_HPO_4_, 22 mM KH_2_PO_4_, 86 mM NaCl, 1 mM MgSO_4_·7H_2_O) and centrifuged at 190× *g* for 2 min before being washed with M9 buffer twice. The washed *C. elegans* were added to 1 mL of RiboEx (GeneAll Biotechnology, Seoul, Korea) and subjected to a gentle vortex for 30 s before being incubated for 30 min at room temperature. Lysed *C. elegans* were frozen at −70 °C overnight and homogenized with 0.5 mM Glass Beads (BioSpec Products, Bartlesville, OK, USA) for 3 min using TissueLyser II (Qiagen). RNA was extracted from homogenized *C. elegans* according to the manufacturer’s instructions. Extracted RNA (approximately 1–3 μg) from worms was transcribed to cDNA using a TOPscript RT DryMIX (Enzynomics, Seoul, South Korea). The amplification of cDNA was conducted using N-Taq DNA polymerase (Enzynomics), which subjected the samples to an initial denaturation at 95 °C for 5 min and 23–32 cycles of denaturation at 95 °C for 10 s, annealing at 60 °C for 15 s and elongation at 72 °C for 30 s. The PCR product was subjected to 1% (*w*/*v*) agarose gel electrophoresis and visualized by staining with ethidium bromide. For the real-time PCR, the samples were reacted using Rotor-Gene Q (Qiagen, Hilden, Germany) to measure the amplification of cDNA using SYBR green (SG, Enzynomics). All experiments included three replicates to ensure statistical significance, and each independent experiment was repeated three times. The relative quantification of gene expression was performed using the comparative threshold cycle (*C*_t_) method. The *C*_t_ value is defined as the point where a statistically significant increase in the fluorescence is observed. The 5′ forward and 3′ reverse complement PCR primers for amplifying each gene were: Act-1 (forward 5′-CCA AGA GAG GTA TCC TTA CC-3′, reverse 5′-CTT GGA TGG CGA CAT ACA TG-3′), zoo-1 (forward 5′-ATT CGG TGG GAC AGT TGG TC-3′, reverse 5′-CGG GTC TAT GGA ACG ATG GG-3′).

### 2.3. Antibacterial Activities of EcN in the Intestine of C. elegans

To prepare for the bacterial colonization assays, worms were age synchronized by bleaching and embryos were incubated on the NGM plates containing *E. coli* OP50 until they reached the L4 stage. Worms were grown on the NGM plates containing GFP (Green Fluorescence Protein)-labeled EPEC for 48 h after being fed *E. coli* OP50 and EcN for 48 h. EPEC was cultured overnight in LB (Luria- bertani broth) broth containing 1% mannose at 37 °C under shaker conditions. Ten worms were picked and placed in 2 mL tubes containing 25 mM levamisole for paralysis and inhibition of pharyngeal pumping. We added LM buffer (25 mM levamisole in M9 buffer) containing 100 μg/mL gentamicin before these tubes were incubated for 45 min to remove surface bacteria. After this, worms were washed in LM buffer before being washed twice more to remove any remaining bacteria and antibiotic. The washed worms were incubated with 1% Triton X-100 and then mechanically disrupted using a motor pestle for 3 min. The lysates of worms were diluted in M9 buffer and spread on LB agar containing 50 μg/mL ampicillin and incubated overnight at 37 °C. The colonies were quantified and used to calculate the number of bacteria per nematode. The intestinal GFP-labeled EPEC was examined using a fluorescence microscope (Eclipse Ts2R, Nikon, Tokyo, Japan). The multi Gauge V3.0 (Fujifilm Life Science, Cambridge, MA, USA) program was used to measure intestinal GFP–EPEC density.

### 2.4. Staining with Nile Red and Oil Red O

Nile Red (Sigma-Aldrich) was dissolved in DMSO to produce a 0.5 mg/mL stock solution, which was stored at −20 °C. The stock solution was freshly diluted with 40% isopropanol to have a concentration of 3.0 μg/mL. Worms washed with M9 buffer twice were fixed with 40% isopropanol containing 0.01% Triton X-100 and then permeabilized using 0.1% Triton X-100 in M9 buffer overnight at 4 °C. Fixed worms were washed with M9 buffer and stained with a working solution of Nile Red for 30 min at RT (Room temperature) with gentle rocking. After staining, worms were washed with M9 buffer three times. Nile Red fluorescence was also examined using the fluorescence microscope (Eclipse Ts2R, Nikon, Tokyo, Japan). As described above, fixed worms were incubated with 60% isopropanol for 15 min at RT with gentle shaking. After aspiration with isopropanol, worms were added to the Oil Red O (Sigma-Aldrich) working solution. The Oil Red O working solution was made using the following steps. A stock solution (0.5 g of Oil Red O in 100 mL of isopropanol) was mixed with dH_2_O at a ratio of 3:2 before the mixed solution was filtered using a syringe (0.4 μM pore size). Stained worms were rinsed with 60% isopropanol for 10 min. After this, the worms were washed with M9 buffer containing 0.01% Triton X-100. Sections were examined using the fluorescence microscope (Eclipse Ts2R, Nikon, Tokyo, Japan). We used the Multi Gauge V3.0 program to measure Oil Red O and Nile Red density. To prevent offspring production, experiments were conducted on NGM plates containing FUdR (50 µM).

### 2.5. Intestinal Barrier Function Assay (Smurf Assay)

Animals were raised following the above-described method. Treatment with EPEC or EcN was described in the result section. Treated worms from the NGM plates were further cultured in the liquid media of UV-killed *E. coli* OP50 bacteria mixed with blue food dye (TCI, FD&C Blue #1, B0790, Portland, OR, USA, 5.0% *w/v* in NGM liquid solution) for 3 h. To prevent offspring production, experiments were conducted on NGM plates containing FUdR (50 µM). Animals were washed in M9 buffer until the blue color of the dye was not visible before they were anesthetized in M9 buffer containing 25 mM levamisole. After this, the worms were analyzed on the slide for the presence of blue food dye in the body cavity using Eclipse Ts2R microscope (Nikon, Tokyo, Japan) under 20× magnification. Data were analyzed using GraphPad Prism.

### 2.6. Intestinal Injury Models in Mice

For chemical-induced colitis, six-week-old female mice were pretreated twice with a vehicle or 1 × 10^9^ EcN per mouse over six days (*n* = 15). For EPEC (E2348/69) infection, C57BL/6J mice were allowed to acclimate for 7 days. All mice were individually housed in ventilated cages with free access to food and water. EPEC was grown to stationary phase in LB broth. Aliquots of the broth culture (1 mL) were centrifuged and the bacterial pellet was suspended in 1.25 mL phosphate-buffered saline (PBS). A suspension containing approximately 1 × 10^9^ E2348/69 cells in 200 µL of PBS was introduced into the mice by gavage with a curved needle 4 cm in length with a steel ball at the tip. Control animals received 200 µL sterile PBS. Over the course of infection, the mice were observed daily to assess activity levels and water intake, and body weight was measured. On the third day following infection, the animals were sacrificed after anesthetization by isoflurane inhalation and intestinal tissues were processed for further analysis.

### 2.7. Fluorometric Assay of FITC-Conjugated Dextran In Vivo

Intestinal permeability was evaluated in different groups of mice by using an orally administered permeability marker, FITC-conjugated dextran, dissolved in water (4 kDa, 50 mg/mL, Sigma-Aldrich). Blood serum was collected 3 h later. The serum concentration of FITC-conjugated dextran was determined by using a plate reader (SpectraMax M2e, Molecular Devices, Sunnyvale, CA, USA). The sample emission spectrum (535 nm) was recorded after excitation at 490 nm. The concentration of FITC-conjugated dextran in serum was determined by applying a FITC-dextran standard curve.

### 2.8. Immunohistochemistry

Before being subjected to immunostaining, the formalin-fixed paraffin-embedded tissues from mice intestines were cut (5 μm), deparaffinized, and rehydrated. Tissue slides were heated in 10 mM sodium acetate (pH of 6.0) for 5 min at 121 °C for antigen retrieval before being bathed in a 0.3% H_2_O_2_–PBS solution for 15 min at room temperature in the dark to quench endogenous peroxidase. After the samples were washed with Tris-HCl/Tween 0.5%, tissue sections (5 μm) were blocked with 5% fetal bovine serum in PBS, incubated in a 1:250 dilution of the primary antibodies overnight at 4 °C and repeatedly washed using PBS. The primary antibodies that were used include anti-claudin-3 antibody (1:250 dilution; Santa Cruz Biotechnology, Santa Cruz, CA, USA) and anti-ZO-1 antibody (1:250 dilution, Santa Cruz Biotechnology, Santa Cruz, CA, USA). Samples were incubated with horseradish peroxidase-conjugated anti-mouse or anti-rat IgG antibodies (1:250 dilution; Enzo Life Sciences, Inc., Farmingdale, NY, USA) for 2 h at room temperature before being subjected to repeated washing with PBS. The bound antibodies were identified using freshly prepared substrate buffer (0.05% diaminobenzidine (DAB; Sigma-Aldrich Chemical Co.), 0.015% H_2_O_2_ in PBS) for 10 min. After a final wash in PBS and distilled water, the slides were counterstained with Mayer’s hematoxylin for 5 min before being dehydrated in graded alcohols (50%, 70%, 80%, 90%, 95%, 100%, and 100%). Sections were examined at various magnifications using a fluorescence microscope (Eclipse Ts2R, Nikon, Tokyo, Japan).

### 2.9. Alcian Blue (AB) Staining of Murine Tissues

For AB staining, sections were deparaffinized in xylene, rehydrated in ethanol, and placed in distilled water for 5 min. AB 8GX (Alcian blue 8GX, Biosesang, Seoul, Korea) was applied to the sections for 30 min at RT, which was followed by a 2 min wash in running tap water. Finally, they were counterstained with Nuclear Fast Red. Stained sections were dehydrated very quickly in two changes of 95% ethyl alcohol, which was followed by two changes of absolute alcohol. This was cleaned using xylene and mounted in synthetic mountant (ThermoFisher Scientific, Seoul, Korea).

### 2.10. Statistical Analysis

Statistical analyses were performed using GraphPad Prism v. 5.01 (La Jolla, CA, USA). For comparative analysis of two groups of data, Student’s *t* test was performed. For comparative analysis of multiple groups, data were subjected to analysis of variance (ANOVA) with Newman–Keuls method as a post hoc ANOVA assessment.

## 3. Results

### 3.1. Antagonistic Actions of EcN against the Gut Colonization of EPEC in the Worms

**Figure 1:**
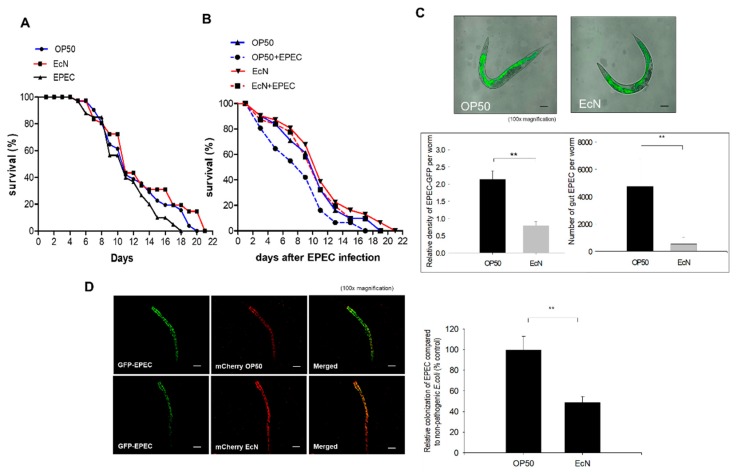
Effect of *Escherichia coli* strain Nissle 1917 (EcN) on enteropathogenic *E. coli* (EPEC)-infected worms. (**A**) For lifespan assays in the presence of each bacterium, presynchronized L4 worms were grown on the *E. coli* OP50, EcN, and EPEC lawns (without tryptophan) for 48 h. To prevent the progeny of the produced offspring, worms were transferred to a new nematode growth medium (NGM) plate every day. The survival of the worms was assessed by counting living worms daily. (**B**) For lifespan assays to measure the impact of EcN pretreatment, presynchronized L4 worms were grown on *E. coli* OP50 or EcN lawn for 48 h and were further grown on NGM plates containing OP50 or EPEC in tryptophan (2 mg/mL) for 48 h. The survival of the worms was assessed by counting living worms daily after EPEC infection (day = 1). (**C**) Fluorescence microscopic observation was used to visualize the amount of EPEC (the upper panel, original magnification ×100; scale bar(s), 100 μm). The experiments were performed as follows. Wild-type (N2) worms were exposed to GFP-expressing EPEC for 48 h on NGM plates containing tryptophan (2 mg/mL) after being fed *E. coli* OP50 and EcN for 48 h. The lower graphs indicate the quantitative analysis of EPEC in the gut. Asterisks represent significant differences between the groups (** *p* < 0.01). All data represent three independent experiments. (**D**) Competitive bacterial colonization in the worm gut. Worms were preincubated with mCherry-labeled nonpathogenic *E. coli* (OP50 or EcN, red) for 24 h before being infected with GFP-labeled EPEC (without tryptophan, green color) for 24 h. The right panel shows the quantification of GFP-labeled EPEC in the gut. The results are shown as mean values ± SEM (Standard error of the mean) (*n* = 18), with the asterisk representing significant difference from the OP50-exposed control group (** *p* < 0.01). Each result is representative of three independent experiments.

Instead of the mammalian exposure-based assessment of bioactive food components, the model of *C. elegans* was used for prolonged luminal exposure due to its short lifespan. The wild-type *C. elegans* (N2) was examined to address the actions of probiotic bacteria EcN against enteropathogenic *E. coli* (EPEC). EPEC is increasingly associated with the pathogenic stress on the intestinal surface of patients with chronic diseases, such as inflammatory bowel disease (IBD) and colorectal cancer [14,15,16,17]. In the present exposure model, EPEC shortened the lifespan of *C. elegans,* compared to that of uninfected hosts (Figure 1A). In contrast, EcN treatment slightly elongated the survival of *C. elegans* (Figure 1A). Based on an assumption that EcN could protect against EPEC infection, we assessed the effects of pretreatment with EcN on the survival of EPEC-infected worms. Moreover, since tryptophan plays crucial roles in bacterial pathogenesis [28], it was supplemented to boost the virulence of EPEC in *C. elegans* model (Figure 1B). Pretreatment with EcN significantly improved the survival of worms in response to an EPEC infection (Figure 1B). The early pathogenesis of EPEC is related to the formation of attaching and effacing (A/E) lesions via actin cytoskeletal rearrangement in human intestinal epithelial cells, which facilitates bacterial binding and subsequent invasion [23]. Based on this known mechanism, we tested whether the protective actions of EcN were mediated by interference with the intestinal colonization of EPEC in the worm gut. *C. elegans* was infected with EPEC after being grown on the lawn of *E. coli* OP50 or EcN, respectively. The luminal levels of EPEC colonization were quantified by measuring GFP-labeled EPEC in the worm intestines. EcN pretreatment significantly reduced the accumulation of EPEC in the gut of *C. elegans* compared to the control group (*E. coli* OP50) (Figure 1C). Although the nonpathogenic OP50 was overwhelmed by EPEC, EcN showed resistance to the pathogen colonization-induced replacement (Figure 1D), indicating that EcN was more competitive with EPEC in the intestinal colonization than the control bacteria OP50.

### 3.2. Improved Gut Permeability by EcN Treatment in Response to EPEC Infection

**Figure 2:**
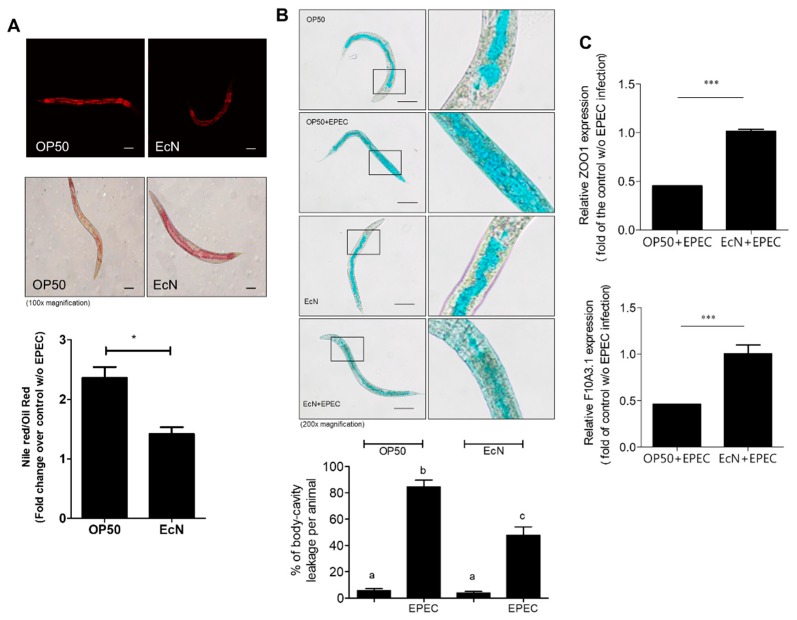
Effect of EcN on gut barrier in worms. (**A**) Wild-type (N2) worms were exposed to EPEC (without tryptophan) for 24 h after being fed *E. coli* OP50 and EcN for 24 h. The collected worms of each group were stained with Nile Red (the upper panels) or Oil Red O (the middle panels). The ratios of Nile Red to Oil Red O were calculated to determine the permeability of the gut barrier of *Caenorhabditis elegans* (×100; scale bar(s), 100 μm) (the lower graph). Data represent mean ± SD (standard deviation) (*n* = 10; * *p* < 0.05 compared with the vehicle control group). (**B**) The gut permeability was further verified using the Smurf assay. Presynchronized wild-type L4 worms were exposed to EPEC (without tryptophan) for 24 h after being fed *E. coli* OP50 and EcN for 24 h. The control groups (OP50 and EcN) were exposed to *E. coli* OP50 for 24 h instead of EPEC treatment (original magnification ×200). The lower graph shows % of body-cavity leakage per animal. Different letters over each bar represent significant differences between groups (*p* < 0.05). (**C**) Quantification of ZOO-1 (human ZO-1 homologue) and F10A3.1 (human Claudin homologue) mRNA was performed to confirm the barrier function using real-time qPCR. After *C. elegans* were fed different types of *E. coli* strain, such as OP50 and EcN bacteria for 24 h, they were exposed to EPEC (without tryptophan) for 3 h. Asterisk represents significant differences between the groups (*** *p* < 0.001). Each result is representative of three independent experiments.

Mechanistically, it is well known that EPEC-induced cytoskeletal rearrangement leads to disruption of the gut epithelial microvilli and barrier [12]. Thus, we investigated the effects of EcN on EPEC-induced collapse of the worm gut barrier by measuring the permeability as the key parameter for evaluating the gut barrier integrity. The measurement of permeability was based on staining using Nile Red and Oil Red O (Figure 2A). As a lipophilic dye, Nile Red was used to measure the fat under the intestinal epithelial layer while Oil Red O was used for counterstaining the major lipid storage region in the nematodes [24,29]. Of note, the treatment with EcN significantly reduced the relative permeability of an EPEC-infected gut compared to the OP50 treatment group (Figure 2A). Moreover, the barrier integrity was tested using a non-absorbable blue food dye, which was visualized microscopically. Although the majority of the dye stains was observed within the intact gut lumen, EPEC-infected worms displayed spreading of the dye into their body cavity (Figure 2B). However, EcN pretreatment partly counteracted EPEC-induced dye spreading to the body cavity, which supports the barrier-protective action of EcN in the worm gut. Since impaired epithelial TJs cause the loss of epithelial cell integrity and disruption of epithelial polarity, we hypothesized that the TJ could be involved in EcN-induced protection against EPEC-induced barrier disruption. In particular, the levels of ZOO-1 (human ZO-1 homologue) and F10A3.1 (human Claudin homologue), which acted as a representative marker of the TJ, was significantly elevated by EcN exposure (Figure 2C), which supports the evidence of protective actions of EcN against EPEC-induced gut barrier disruption. EcN improved the gut barrier integrity of worms by preferentially enhancing the TJ molecules.

### 3.3. EcN-Improved Gut Integrity-Related Features in the Mammalian Infection Model

**Figure 3:**
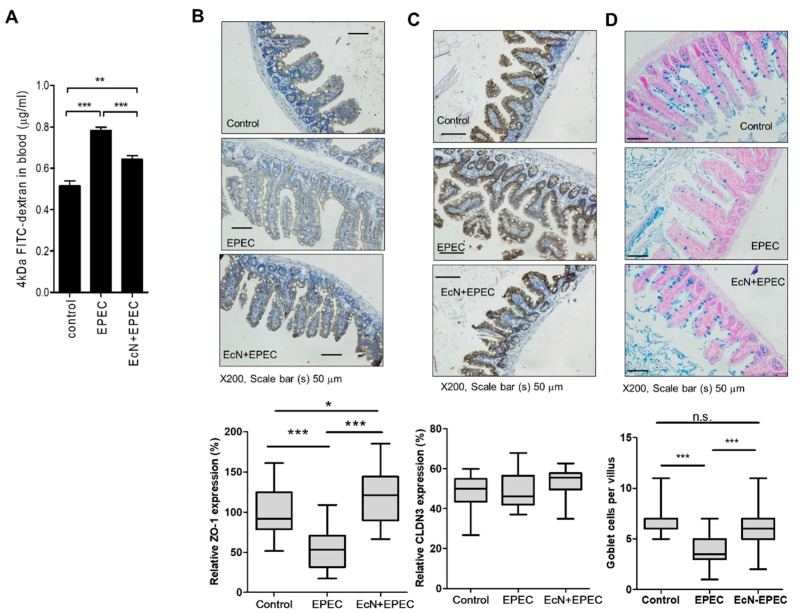
Effect of EcN on gut barrier integrity in EPEC-infected mice. Mice pretreated with EcN (2 × 10^9^ cfu (colony-forming unit) per mouse) were exposed in EPEC-infected mice. (**A**) Serum FITC-dextran levels. (**B**,**C**) Expression of tight junction (TJ) molecules in the colon of mice with colitis. ZO-1 (B) or claudin-3 (C, CLDN3) were analyzed with immunohistochemistry analyses in the colon at day 10 (original magnification ×200; scale bar(s), 50 μm). (**D**) The sections were stained with Alcian blue for the analysis of goblet cells and mucin production (original magnification ×200; scale bar(s), 50 μm). Lower graphs indicate the quantitation of each staining using the HistoQuest analysis software. Asterisks represent significant differences between the groups (* *p* < 0.05, ** *p* < 0.01, *** *p* < 0.001).

EcN treatment-induced gut protection was verified in the mammalian barrier using a murine gut model. In addition to protection against barrier disruption in the worm gut barrier model, the actions of EcN was confirmed in the murine model of EPEC infection. EcN-pretreated mice were orally administered with EPEC, and infection-linked effects on gut barrier integrity were measured. EPEC infection or EcN treatment did not cause significant changes in the colon length of mice (data not shown). However, as a sign of a leaky gut barrier, FITC-dextran can be detected in the serum of mice with gut barrier injury. EPEC infection led to significant increase in serum dextran, which was partly attenuated by treatment with EcN (Figure 3A). Barrier protection by EcN was verified by assessing expressions of junctional molecules (ZO-1 and clauidin-3) of the gut epithelial lining in response to EPEC infection. The histological levels of ZO-1 and claudin-3 are used as the epithelial barrier-associated biomarkers [30]. EPEC infection downregulated the expression of ZO-1, which was counteracted by EcN pretreatment (Figure 3B). In contrast with results in the worm model, the expression of claudin-3 as another biomarker of the gut epithelial barrier was not significantly affected in the mouse infection model. However, the mucus secretion as a biomarker of mucosal barrier was suppressed by EPEC infection (Figure 3C). According to Alcian blue staining, the number of mucin-secreting goblet cells decreased, but they were restored by pretreatment with EcN (Figure 3D). Mucus secretion from the restored population of goblet cells is a crucial defense against microbial access to host cells in the gut.

## 4. Discussion

Extensive experimental evidences on the actions of probiotic bacteria have proven their safety and efficacy for human applications before marketing. The present study assessed a non-mammalian model that can be used for the evaluation of the probiotic actions of EcN. Among the various beneficial actions of probiotic bacteria, we focused on the evaluation of mucoactive properties using the *C. elegans* gut model. Epithelial recognition is crucial in transmitting the danger signals into other parts of the body to trigger the production of defense molecules, which can subsequently recruit the circulating immune cells. The murine-based models have been extensively used to screen valuable barrier-active components from the food or other sources [31,32,33]. However, due to increasing ethical issues and complexity in human extrapolation, the present study performed a *C. elegans*-based assessment of mucoactive probiotic bacteria using EcN as a representative facultative anaerobic bacterium in the market. Compared to the mammalian intestinal barrier with heterogeneous composition of various cellular and matrix components, the gut of *C. elegans* is a highly simple model that does not involve any specialized immune cells. Instead of the specialized immune cells, the gut epithelial lining is the pivotal defense barrier of the animals against the external infections and xenobiotic stressors. The nematode gut model provides an advantageous methodology over the previous mammalian models in assessing the mucoactive bacteria in a specific way. EcN counteracted the infection and epithelial disruption caused by enteropathogenic *E. coli*.

Mechanistically, EcN colonization in the worm elevated the expression of junctional protein ZOO-1, which was also verified in the mammalian infection and colitis model. Although ZO-1 expression was elevated by EcN, claudin-3 levels were not significantly improved by EcN in EPEC-infected mouse gut barrier. In contrast, EcN elevated the expression of both claudin-like molecule (T10A3.1) and ZOO-1 in worms. In terms of mammalian transcription, ZO-1 expression is promoted by specific transcription factors such as AP-1 (activator protein 1) and CREB (cAMP response element binding protein) whereas SP-1 (specificity protein 1) plays crucial roles in claudin-3 transcription [34,35]. Therefore, differential clustering of transcription factors would lead to specific regulation of ZO-1 without affecting expressions of other TJ molecules. Moreover, genetic ablation of ZO-1 level had marginal impacts on expressions of other TJ molecules including claudins and occludin in the epithelial cell layer [36], indicating independent pathways of ZO-1 expression. In the present study, EcN improved expression of ZO-1, which is a crucial scaffold protein for direct linker molecules such as claudins and occludin in the intercellular gap. Therefore, even without changes in expressions of claudins and occludin, reduction in ZO-1 level may cause severe disorganization of the protein complex in response to the ulcerative injury [37]. In the present gut barrier model of worms and mice, EcN-improved ZO-1 would be a potent major mediator of the pharmacological or nutritional intervention of the probiotic bacteria. Some probiotic bacteria, such as *Bifidobacterium bifidum,* are known to strengthen the intestinal epithelial TJ using the epithelial monolayer model [32]. In addition to the pathogen-suppressing action, EcN improved the gut barrier integrity of worms, which was also verified in the murine model. In our present models, the expression of ZO-1, the key junctional molecule of the epithelial barrier, was upregulated by EcN treatment although the exact bacterial mediator was not identified. Moreover, the goblet cell-derived mucin production was highly elevated by EcN treatment. In addition to the junctional integrity, optimized differentiation into goblet cells or mucin production was improved by EcN in the mammalian model. Therefore, more sophisticated mechanistic evidences of these integrated beneficial actions of EcN should be investigated in future studies.

In addition to the direct actions of bacterial mediators against the barrier disruption, nonbacterial factors may contribute to EcN-induced protection in the present gut models. The gut barrier integrity is susceptible to inflammatory insult in mammals. In particular, pro-inflammatory cytokines including tumor necrosis factor alpha and interleukin 1 beta are markedly elevated in patients with inflammatory gut distress [38,39] and are positively involved in the disruption of the epithelial barrier [40,41]. It can be thus hypothesized that EcN attenuates the detrimental actions of pro-inflammatory cytokines and barrier disruption during EPEC infection. However, compared to the early disruption of TJ after one day of EPEC infection, the neutrophil recruitment and pro-inflammatory cytokine production are evident after 5–10 days infection in mice [42,43]. Therefore, inflammatory insults would be a marginal factor in triggering early barrier disruption during EPEC infection in the murine model.

Using the worm gut model, EcN was proven to compete with pathogenic bacteria in colonization on the mucosal surface. In addition to evaluating the counteracting actions against EPEC, this method can be applied to assess whether the probiotic bacteria potentially compete with other gut microbes, including commensal bacteria. Although the pathogen-competing EcN can be beneficial for health, it can also interfere with the colonization of beneficial bacteria, such as OP50 in the worms. A recent report demonstrated that probiotics perturb microbiota colonization rather than aid microbiota recovery back to baseline after antibiotic treatment in humans [44]. It implicates that EcN can interfere with other commensal bacteria rather than cooperating with the residential bacteria, which could be another concern with regards to the application of unknown bacteria in the normal microflora. Evaluation of the bacterial competition using the *C. elegans* model would provide precise, easier, and comprehensive insights into the risk and benefit of the candidates with minimal interference with the stable gut microbial ecology.

Other than the mammalian exposure model, the in vitro epithelial monolayer model using enterocytes has become most popular for testing the barrier permeability by measuring the transepithelial resistance [32]. However, the simple epithelial monolayer does not give crucial information on the spatial impact of the food components on the three dimensional monolayer in the gut. Using the worm gut model, we can easily estimate the in vivo dynamic impact of the microbes on the gut barrier integrity in the different parts of mucosal site of the gut lining. Instead of the two-dimensional model of the epithelial monolayer, a gut organoid could be used in an alternate three-dimensional assessment of bacterial actions in the lumen of the organoid [45,46]. Moreover, the gut organoid model contains the crypt progenitor-derived architecture, which provides a more realistic environment of dynamic gut lumen for gut microbiota. However, it is hard to maintain the gut luminal exposure to bacteria in the inner side of the organoid without leaking. The present nematode model contains the stem cell population in the gut and is highly cost effective with an easier bacterial application methodology to screen a variety of barrier-active drugs and biologics and their behavior in both longitudinal and horizontal ways in the gut lumen. Taken together, worm-based evaluations of dietary probiotic microbes could be a useful alternative model of the mammalian gut barrier or the in vitro epithelial model due to their excellent performance on the risk or benefit evaluation.
